# LIM Tracker: a software package for cell tracking and analysis with advanced interactivity

**DOI:** 10.1038/s41598-022-06269-6

**Published:** 2022-02-17

**Authors:** Hideya Aragaki, Katsunori Ogoh, Yohei Kondo, Kazuhiro Aoki

**Affiliations:** 1 Innovation and Core Technology Management, Olympus Corporation, Kuboyama 2-3, Hachioji, Tokyo 192-8512 Japan; 2 Quantitative Biology Research Group, Exploratory Research Center on Life and Living Systems (ExCELLS), National Institutes of Natural Sciences, 5-1 Higashiyama, Myodaiji-cho, Okazaki, Aichi 444-8787 Japan; 3 Division of Quantitative Biology, National Institute for Basic Biology, National Institutes of Natural Sciences, 5-1 Higashiyama, Myodaiji-cho, Okazaki, Aichi 444-8787 Japan; 4 Department of Basic Biology, School of Life Science, SOKENDAI (The Graduate University for Advanced Studies), 5-1 Higashiyama, Myodaiji-cho, Okazaki, Aichi 444-8787 Japan

**Keywords:** Cell biology, Computational biology and bioinformatics

## Abstract

Cell tracking is one of the most critical tools for time-lapse image analysis to observe cell behavior and cell lineages over a long period of time. However, the accompanying graphical user interfaces are often difficult to use and do not incorporate seamless manual correction, data analysis tools, or simple training set design tools if it is machine learning based. In this paper, we introduce our cell tracking software “LIM Tracker”. This software has a conventional tracking function consisting of recognition processing and link processing, a sequential search-type tracking function based on pattern matching, and a manual tracking function. LIM Tracker enables the seamless use of these functions. In addition, the system incorporates a highly interactive and interlocking data visualization method, which displays analysis result in real time, making it possible to flexibly correct the data and reduce the burden of tracking work. Moreover, recognition functions with deep learning (DL) are also available, which can be used for a wide range of targets including stain-free images. LIM Tracker allows researchers to track living objects with good usability and high versatility for various targets. We present a tracking case study based on fluorescence microscopy images (NRK-52E/EKAREV-NLS cells or MCF-10A/H2B-iRFP-P2A-mScarlet-I-hGem-P2A-PIP-NLS-mNeonGreen cells) and phase contrast microscopy images (Glioblastoma-astrocytoma U373 cells). LIM Tracker is implemented as a plugin for ImageJ/Fiji. The software can be downloaded from https://github.com/LIMT34/LIM-Tracker.

## Introduction

Cell migration plays important roles in various processes, including embryonic development, cell differentiation, immune response, regeneration, and tumor invasion^1–5^. To better understand the regulatory mechanisms of cell migration, it is essential to have a time-lapse imaging tool to observe the continuity of cell behavior and cell lineages over a long period of time. There are currently technical limitations to long-term live-cell time-lapse imaging. Time-lapse imaging requires imaging at sufficient frequency to track a population of cells with irregular positions and morphological changes over time. For image analysis with high reproducibility, an image with a high S/N ratio is required, but, in many cases, it is difficult to obtain an image with a high S/N ratio at a high sampling rate because of phototoxicity in living cells or individuals^6,7^. Recent advances in microscope technology have made this possible, but it is still difficult to analyze the enormous amount of image data obtained after image acquisition with high accuracy. To investigate these images, it is essential to develop advanced analytical software, including high-precision segmentation methods for extracting information on the position and morphology of individual cells, and tracking methods for quantifying the dynamics of cells, among other features. However, at present there is a scarcity of tracking software with excellent usability and versatility suitable for long-term time-lapse experiments.

One of the most significant factors making tracking software impractical is that the microscopically imaged targets vary in appearance, have low S/N ratios, and exhibit many visual patterns, such as a variety of cell morphologies. Because cell images have various visual characteristics, there has been no algorithm that can handle a wide range of cell types and various microscopy modalities, such as fluorescence microscopy and phase contrast microscopy. Recognition and tracking of wide ranges of patterns is therefore technically challenging, which makes it difficult to develop universal tracking software. In recent years, with the advancement of deep learning (DL) recognition technology, it has become possible to perform recognition with higher accuracy than before^8–11^, but even so, it is still impossible to fully automate recognition and tracking, and to prevent a certain number of processing errors from occurring. To solve these problems and improve the tracking work efficiency, it can be effective to prepare an appropriate operation system in which a user can quickly confirm an error from a large amount of data and can efficiently correct the error on the assumption that a certain number of erroneous trackings may occur. However, since tracking processing requires multidimensional (x, y, time, channel) analysis of a large amount of image data, software design is complicated, and it is not easy to realize software with high usability while ensuring high-speed processing and response.

Automatic tracking platforms established to date include “TrackMate”^12^, “CellProfiler”^13^, “MMHelper”^14^ and “Usiigaci”. TrackMate can perform recognition and tracking with simple wizard-based operations, but the data display for checking the tracking results is fixed and lacks interactivity, making it difficult to find the data on the spot of interest among a large amount of data. Editing trajectories (groups of spots linked between frames) can only be done on the cell lineage screen (TrackScheme), but it is difficult to determine the correctness of the trajectory from the lineage diagram alone, and the operation is complicated, especially when the number of targets increases. Manual correction methods to efficiently correct errors in tracking results are also not sufficiently supported. Another automatic tracking platform, CellProfiler, uses a pipeline method by combining various processing modules and can handle a large number of multi-channel images. Finally, Usiigaci has a powerful DL recognition function that can handle a variety of targets including stain-free images. However, both of them lack usability because they cannot be operated with a graphical user interface that enables effective interaction with the user, and they do not provide a means to check the validity of processing results or to correct them. In addition, the former requires users to have specialized knowledge of image processing and analysis, while the latter is difficult to use unless the user has a certain level of programming knowledge.

We developed LIM Tracker to solve some of the above problems. LIM Tracker is a tracking software that provides researchers with a highly versatile tracking solution with excellent usability. Our software is equipped with a tracking function that allows a flexible combination of manual and automatic tracking approaches. It also incorporates a highly interactive and interlocking data visualization tool that displays analysis result in real time, and together with a simple and flexible correction method, reduces the burden of tracking work. Table 1 shows a comparison of the functions and performance of LIM Tracker with those of the above existing software. The performance is evaluated based on the index used in the ISBI Cell Tracking Challenge^6^ (Supplementary S1.1). LIM Tracker is designed to be versatile and can be applied to a wide range of tracking applications, from cell lineage analysis to particle tracking of organelles, at the tissue, cellular, and molecular levels. Researchers can streamline the tracking process, which used to take an enormous amount of time and effort, and use it to promote their research.

**Table 1:** Comparison of LIM Tracker with those of the existing software.

		TrackMate (ImageJ/Fiji)	CellProfiler (Broad Institute)	MMHelper (Univ. of Exeter)	Usiigaci (OIST)	LIM Tracker
**Comparison of the function**						
Tracking function	Link-type tracking	Yes	Yes	Yes	Yes	Yes
	Sequential tracking	No	No	No	No	Yes
	Manual tracking	Limited^**a**^	No	No	No	Yes
Interactive real-time data linkage display		No	No	No	No	Yes
ROI editing function		Yes^**b**^	No	No	No	Yes^**c**^
Trajectory editing function		Limited^**d**^	No	No	No	Yes
Recognition function (non-DL)		Yes	Yes	Yes	No	Yes
DL recognition function	Recognition function	Yes	No	No	Yes	Yes
	Training function (integrated UI including annotation)	No	No	No	Limited^**e**^	Yes
Performance on ISBI Cell Tracking Challenge^6^ “PhC-C2DH-U373” dataset (Fig. 5c)						
Recognition accuracy (SEG) ^**f**^		0.68^g^	N/A^**h**^	N/A ^**i**^	0.89^**j**^	0.93^k^
Detection accuracy (DET) ^**l**^		0.93	N/A	N/A	0.97	0.98
Tracking accuracy (TRA) ^**m**^		0.93	N/A	N/A	0.97	0.98

a, Cannot be combined with other tracking functions. b, The region shape cannot be set. c, The region shape can also be freely set. d, It only works for a very small number of targets. e, A Python script file is provided, and executed by command line operations. f, l, m, The evaluation index is the one used in the ISBI Cell Tracking Challenge^6^, and is calculated using a publicly available evaluation program. g, Use the DL recognition function “Stardist (Stardist detector custom model)”^23^. Since it has no training function, it creates trained weight files based on command line operations. h, i, It did not have a high-performance recognition function and could not be recognized. j, Use the DL recognition function “Mask R-CNN”^25^. The included Python script is used to create a trained weight file based on command line operations. k, Use the DL recognition function “Mask R-CNN”^25^, and the training function (integrated UI including annotation) enables efficient training with simple operations.

## Method

### Plasmids

The cDNA of Histone 2B (H2B)-iRFP-P2A-mScarlet-I-human Geminin (hGem)-P2A-PIP-tag-NLS-mNeonGreen was synthesized with codon optimization for human by FASMAC into the vector plasmid pUCFa (FASMAC). H2B-iRFP, hGem-mScarlet-I, and PIP-tag-NLS-mNeonGreen are a nuclear marker, S/G_2_/M marker^15^, and G_1_/G_2_/M marker^16^, respectively. The cDNA was further subcloned into a pCSII-based lentiviral vector^17^, pCSIIpuro-MCS, generating pCSIIpuro-H2B-iRFP-P2A-mScarlet-I-hGem-P2A-PIP-tag-NLS-mNeonGreen (sequence and plasmid map are available at the following link: https://benchling.com/s/seq-VNv4wwUusibodKnqVv9f). psPAX2 was a gift from Dr. D. Trono (Addgene plasmid #12,260)^18^. pCMV-VSV-G-RSV-Rev was a gift from Dr. H. Miyoshi (RIKEN, Japan). pCSIIpuro-MCS was a gift from Dr. M. Matsuda (Kyoto University).

### Cell cultures

NRK-52E/EKAREV-NLS cells were purchased from JCRB (catalog number IFO50480). NRK-52E/EKAREV-NLS cells stably express the genetically encoded ERK FRET biosensor, EKAREV-NLS^19^. NRK-52E/EKAREV-NLS was established as described previously^20^. The NRK-52E/EKAREV-NLS cells were maintained with Dulbecco’s Modified Eagle Medium (DMEM; ThermoFisher), 10% fetal bovine serum (FBS; Sigma), and 10 μg/mL Blasticidin S (Invitrogen) at 37 °C under 5% CO_2_ with antibiotics. MCF-10A cells were purchased from Horizon Discovery (catalog number HD PAR-003). MCF-10A cell lines were maintained in full growth medium, which consisted of DMEM/F12 (1:1) (Cat#11,330–032, Gibco) supplemented with 5% horse serum (Cat#16,050–122, Invitrogen), 10 mg/ml insulin (Cat#12,878–44, Nacalai Tesque), 0.5 mg/ml hydrocortisone (Cat#1H-0888, Invitrogen), 100 ng/ml cholera toxin (Cat#101B, List Biological Laboratories), 20 ng/ml hEGF (Cat#AF-100–15, PeproTech), and 1% penicillin/streptomycin (Cat#26,253–84, Nacalai Tesque) at 37 °C under 5% CO_2_ with antibiotics. MCF-10A/H2B-iRFP-P2A-mScarlet-I-hGem-P2A-PIP-NLS-mNeonGreen cells were established through lentivirus-mediated gene transfer into the parental MCF-10A cells. In brief, the lentiviral pCSIIpuro vectors were transfected into Lenti-X 293 T cells (Clontech) together with the packaging plasmid psPAX2 and pCMV-VSV-G-RSV-Rev by using the linear polyethyleneimine “Max” MW 40,000 (Polyscience). After 2 days, MCF-10A parental cells were cultured in the virus-containing media in the presence of 8 μg/mL polybrene for 3–4 h. Two days after infection, the cells were selected by treatment for at least 1 week with 1.0 μg/ml puromycin (InvivoGen). Bulk populations of selected cells were used in this study.

### Fluorescence time-lapse imaging

The NRK-52E/EKAREV-NLS cells were seeded at a density of 2.0 × 10^4^ cells/cm^2^ on glass-bottomed dishes (IWAKI). One day later, time-lapse imaging was performed. The culture medium was replaced with FluoroBrite (ThermoFisher), 5% FBS (Sigma), and 1 × Glutamax (ThermoFisher) 3–6 h before starting the time-lapse imaging. For imaging NRK-52E/EKAREV-NLS cells, a wide-field epi-fluorescence microscope was used with an inverted microscope (IX81; Olympus) equipped with a CCD camera (CoolSNAP K4; Roper Scientific) and an excitation light source (Spectra-X light engine; Lumenncor). Optical filters were as follows: an FF01-438/24 excitation filter (Semrock), an XF2034 (455DRLP) dichroic mirror (Omega Optical), and two emission filters [FF01-483/32 for CFP and FF01-542/27 for YFP (Semrock)]. Images were acquired every 20 s (the exposure time was 100 ms) with binning 8 × 8 on MetaMorph software (Molecular Devices) with an IX2-ZDC laser-based autofocusing system (Olympus). A × 20 lens (UPLSAPO 20 × , Olympus; numerical aperture: 0.75) was used. The temperature and CO_2_ concentration were maintained at 37 °C and 5% during the imaging with a stage-top incubator (Tokai Hit). The MCF10A/H2B-iRFP-P2A-mScarlet-I-hGem-P2A-PIP-NLS-mNeonGreen cells were seeded on a 96-well glass-bottomed plate (Greiner; Cat# 655,892) as described previously^21^. One day later, the cells were imaged with IXM-XLS (Molecular Devices) equipped with an air objective lens (CFI Plan Fluor 10 × , NA = 0.30, WD = 16 mm and CFI Plan Apochromat Lambda 20 × , NA = 0.75, WD = 1 mm; Nikon), a Zyla 5.5 sCMOS camera (ANDOR), and a SOLA SE II light source (Lumencor). The excitation and fluorescence filter settings were as follows: mNeonGreen, excitation filter 472/30, dichroic mirror 350–488 (R)/502–950 (T), and emission filter 520/35 (Part# 1–6300-0450; Molecular Devices); mScarlet-I, excitation filter 562/40, dichroic mirror 350–585 (R)/601–950 (T), and emission filter 624/40 (Part# 1–6300-0449; Molecular Devices); and iRFP, excitation filter 628/40, dichroic mirror 350–651 (R)/669–950 (T), and emission filter 692/40 (Part# 1–6300-0446; Molecular Devices). Images were acquired every 10 min with binning 2 × 2 on MetaXpress software (Molecular Devices). The temperature and CO_2_ concentration were maintained at 37 °C and 5% during the imaging.

### LIM Tracker overview

**Figure 1:**
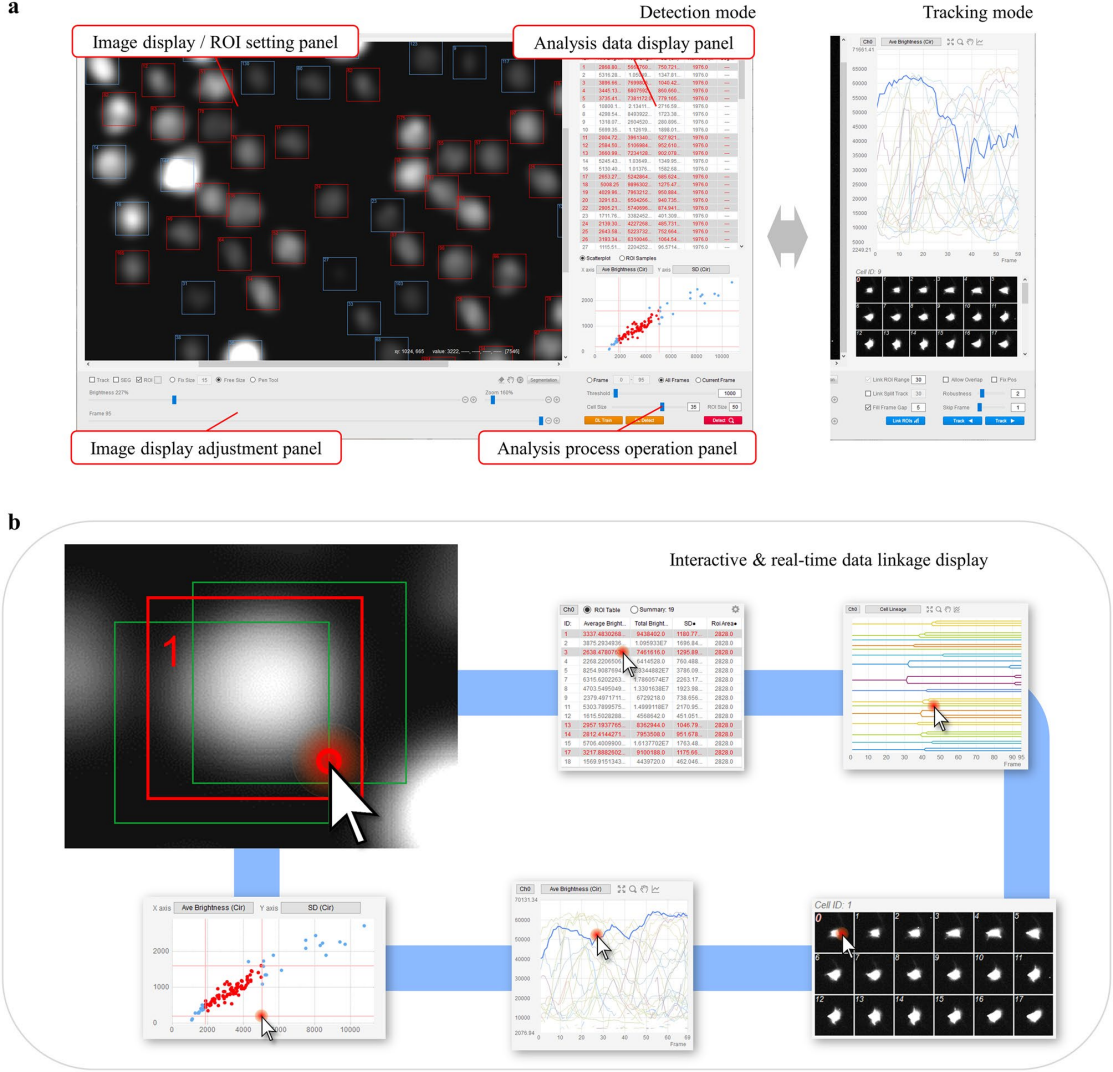
LIM Tracker screen structure. (**a**) LIM Tracker screen structure. The screen is divided into detection mode and tracking mode, and tracking is performed by switching between them. The entire screen is divided into four panels, with image display and ROI setting functions (top left), analysis data display functions (top right), image display adjustment functions (bottom left), and analysis processing operation functions (bottom right) integrated into each panel. (**b**) The ROI and each data display item (list, graph, scatter plot, montage image, cell lineage) are linked, and the display is updated in real time when there is a change in the ROI status. Each item in the data display responds to mouse clicks, and all data related to the selected item are instantly highlighted.

This section describes the unique features of our cell analysis software. The appearance of the screen of this software is shown in Fig. 1a.

#### Combined use of three tracking methods

This software has three different tracking methods.

##### Method 1: link-type tracking

**Figure 2:**
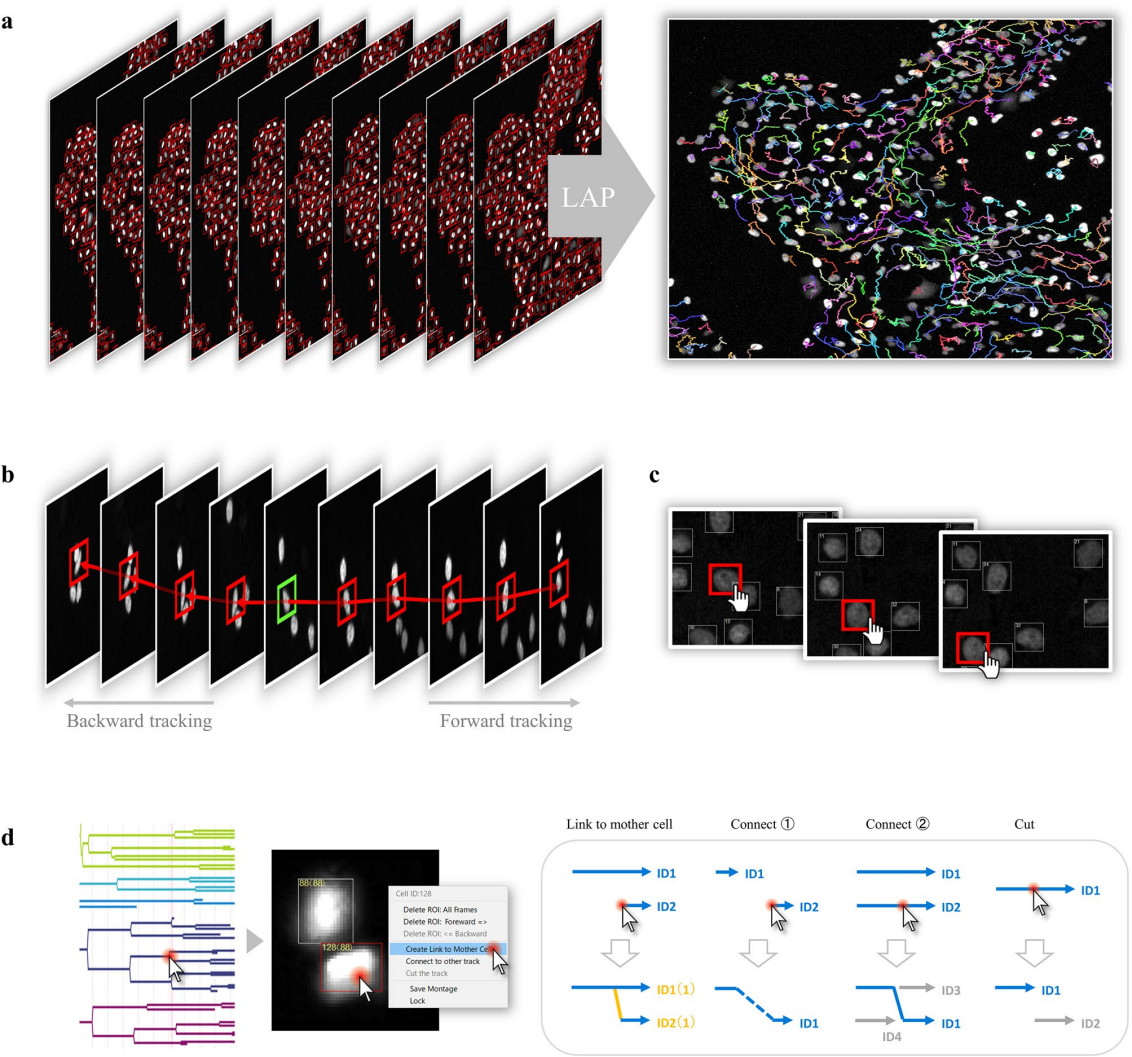
Tracking methods and editing function. (**a**) Overview of the link-type tracking method. First, all image frames are subjected to a recognition process to determine the position and shape of the target, and then ROIs are generated. Then, trajectories are generated by linking ROIs that are determined to be identical between adjacent frames. (**b**) Overview of the sequential tracking method. For a user-selected ROI (indicated by the green), the target region for each frame is searched sequentially based on pattern matching to identify the destination location. Time-series forward tracking as well as backward tracking is possible. (**c**) Overview of manual tracking. It is possible to perform manual tracking and generate trajectories for selected ROIs at any timepoint. (**d**) Overview of the trajectory editing function. By clicking on a point of interest in the cell lineage, the corresponding frame image and ROI are instantly displayed. The user can visually check the positional relationship between the source and target ROIs on the image, and modify the trajectory (link relationship between ROIs) with a simple mouse operation.

The first tracking method is a link-type tracking method (tracking by detection) combining recognition processing and link processing, which is a standard tracking method often adopted in existing tracking software. First, recognition processing is performed on all image frames to recognize the position and region shape of the target in each frame to generate the ROIs (represented by the bounding box in this paper), and then generate the trajectories by linking the ROIs that are determined to be identical between adjacent frames (Fig. 2a). The default recognition processing in this software is assumed to be mainly for fluorescence and emission microscope images, and is equipped with a spot detection algorithm based on a Laplacian of Gaussian (LoG) filter and region formation algorithm based on Marker-Controlled Watershed. (A plugin mechanism is provided for the recognition function, and users can switch to their own algorithm.) In addition, the Linear Assignment Problem (LAP) algorithm proposed by Jaqaman et al. is adopted as the link processing^22^, and by likening the link processing between targets to the optimization processing of the assignment problem, a large number of targets can be linked at high speed (Supplementary S2.1). The tracking performance of this method greatly depends on the recognition accuracy, and while highly accurate tracking processing can be expected for an object such as fluorescence microscope images on which it is relatively easy to identify a target, link processing itself does not function for an object that is difficult to recognize.

##### Method 2: sequential tracking

The second tracking method is a sequential search-type tracking method based on the particle filter framework. In this method, the ROI specified by the user is tracked by sequentially searching for the destination of the ROI and pattern matching it while moving the image frame forward (Supplementary S2.2). Unlike method 1, this method can freely track any region on the screen. Since this method searches based on the similarity of luminance patterns in the area around the target position, it can be applied to targets that are difficult to recognize and track with conventional processing, such as bright-field targets. The ROIs to be tracked can be created by the user directly with the mouse or automatically generated by the above recognition process (Fig. 2b). In addition to tracking in the forward direction, tracking in the reverse direction (i.e., backward in time) can also be performed.

##### Method 3: manual tracking

The third tracking method is manual tracking, which allows users to specify the position of ROIs using the mouse while moving frame by frame (Fig. 2c). The position and size of each ROI can be modified, the shape of the region can be set, and the trajectory can be edited (cut, link, delete) freely using the mouse.

##### Seamless combination of each method and interactive real-time data linkage display

The above three tracking methods can be used in LIM Tracker, but each method has its own advantages and disadvantages. Method 1, the link-type tracking method, can track a large number of targets at high speed, but it will not work unless the target position can be recognized with high accuracy. In contrast, method 2, the sequential tracking method, can track any target with high precision, but it is slow and unsuitable for tracking a large number of targets. In addition, the automatic tracking methods 1 and 2 do not always work perfectly, and unless the target is very easy to track, it is rare that the tracking process is error-free. In contrast, the manual tracking method 3 is 100% accurate, although it has the disadvantages of being time-consuming and laborious. A major feature of LIM Tracker, which is not available in conventional software, is that these three methods can be used in combination a seamless and appropriate manner. By combining each method according to the type and number of targets, recognition accuracy, and other variables, and by complementing the merits and demerits of each method, it is possible to significantly improve the efficiency of tracking. Even when functions for automatic tracking and manual tracking are installed in conventional software, each of these is often functionally independent and does not have a mechanism to freely combine them. In contrast, LIM Tracker has a semi-automatic operation system that allows seamless combination of the three methods. The screen layout and operations have been optimized to minimize unnecessary screen transitions, mode switching, and selection operations, and to make switching between the methods as simple as possible.

As mentioned above, when performing tracking with a combination of methods, if the user cannot see the entire tracking situation and adequately judge the correctness of the tracking results, they will not be able to select the optimal method and the effectiveness will be markedly reduced. In LIM Tracker, there is robust incorporation of interactive operability, analysis in real time, and interlocking of the images, data items, and cell lineage to be displayed on the screen, along with the establishment of visibility and operability lacking in conventional software (Fig. 1b, Supplementary S2.3). These features help users to intuitively grasp the validity of the processing and operation results, and contribute to the efficiency of tracking tasks.

#### Editing function for ROI and trajectory

The user can freely create ROIs on the image at any time by using the mouse. In this software, trajectory editing, such as cutting, connecting, and deleting the trajectories generated by the tracking process, can also be performed directly using the mouse (pull-down menu selection) on the ROIs in the image. In conventional software, a separate screen showing the cell lineage is often prepared, and the trajectory is often edited on the lineage regardless of the ROI displayed on the original image. Since information on the position of individual cells is not visible in the lineage display, it is often difficult to judge whether the trajectory is correct and to correct the link. With this software, users can grasp the overall tracking status by using the cell lineage, and if there is an ROI or trajectory that users want to edit, users can click on the lineage to call up the corresponding frame/ROI on the screen, and perform accurate and efficient editing by directly visually checking the link between the frames of the ROIs located on the image (Fig. 2d).

#### Spot detection and region shape recognition function

The recognition process in this software can detect the spot position of the target, but if necessary, it can also simultaneously recognize the region shape based on the segmentation process, thus quantifying not only the luminance around the spot, but also the change in morphological features of the region. When the segmentation process recognizes the shape of a region, the segmentation results often contain errors (under- or over-segmentation). In many cases, existing software can only modify the segmentation by changing the processing parameters, among other factors, and resegmenting the entire image. However, in LIM Tracker, users can directly add or delete ROIs to the mis-detected portions of the segmentation results using the mouse. In addition, when adding a new ROI, the software has an auxiliary function that automatically generates a region shape by segmenting the ROI region with optimized parameters. It also has a pen tool function, which allows the user to draw freehand curves to create arbitrarily shaped regions.

#### Deep learning recognition function

**Figure 3:**
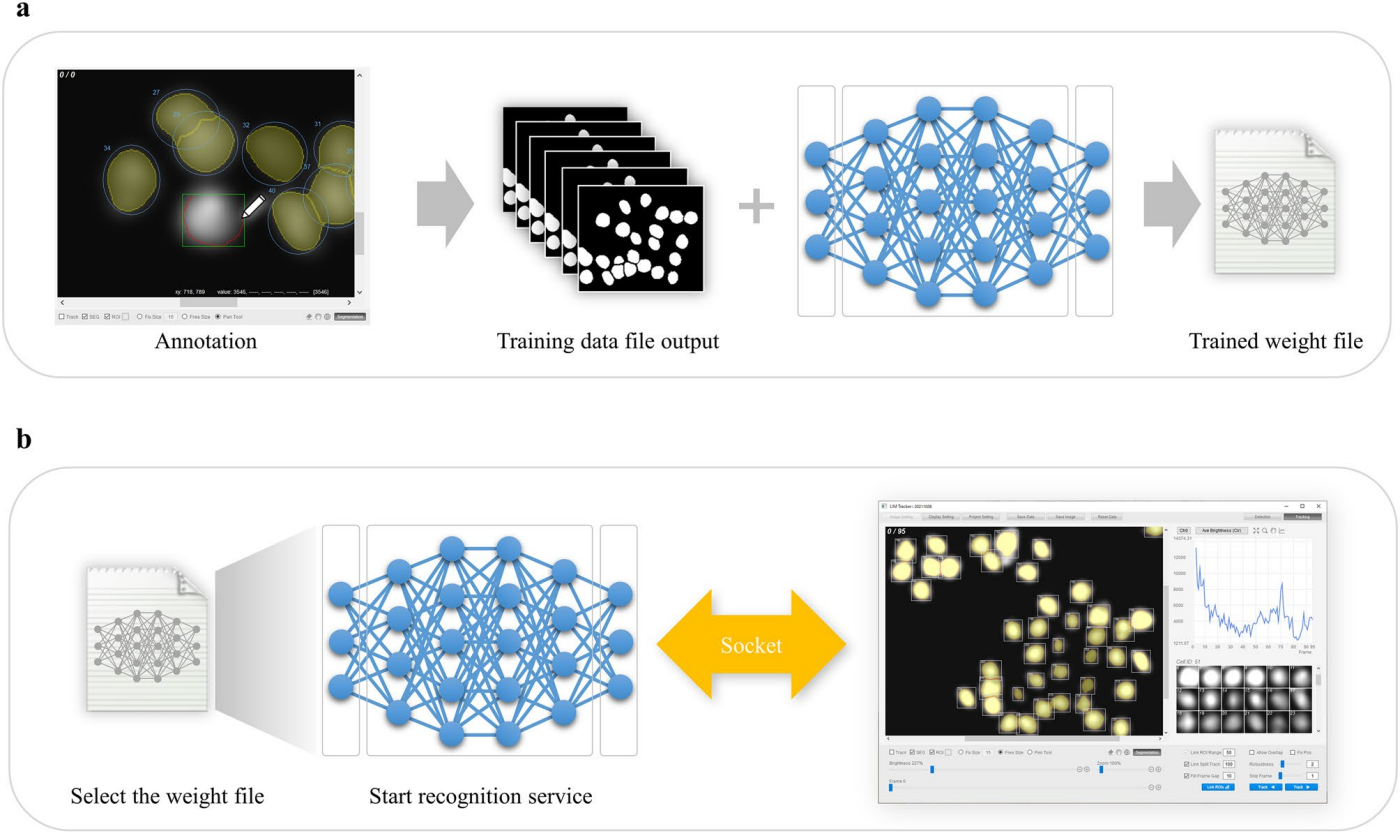
Deep learning training and recognition procedure. (**a**) Training procedure. The software can annotate and create a training dataset file for DL training and can also automatically call an external DL program to train the dataset to generate a trained weight file. (**b**) Recognition procedure. When performing DL recognition, the DL recognition service program can be automatically launched by selecting a weight file on the software, and then the DL recognition process can be executed in cooperation with the recognition service program.

In the analysis of microscopic images, the segmentation technique for recognizing the position and shape of cells has been continuously improved. However, there are still unstained microscopic images (bright-field, phase contrast, DIC images, etc.) that are difficult to identify because of the slight difference between the background and subject areas and the unclear contrast. On the other hand, with the remarkable progress in computer vision and machine learning using DL technology in recent years, it has become possible to analyze microscopic images, including stain-free ones, with high accuracy. However, in reality, it is challenging to perform satisfactory analysis unless the user has a certain level of competence in fields such as Linux and Python programming. In addition, for users to build a recognition process that meets their objectives and obtain optimal recognition results, it is necessary to prepare an environment that allows them to properly learn using datasets created with their own experimental configurations. Unfortunately, there is a lack of software with excellent usability that supports a series of DL operations from the creation of training data (annotation) to training and recognition. In light of the above, we have built into our software a DL operation tool that can be operated efficiently and with as little effort as possible, and that can also be used to easily build a highly accurate recognition process (Fig. 3). Since the annotation tool, namely, the pen tool function mentioned above, is also built-in, users can create training data directly on this software and train it with simple operations to freely build the most suitable recognition process for their own dataset. By combining this DL recognition function with the link-type tracking function, the tracking accuracy can be greatly improved.

Concerning algorithms, there is rapid technological progress in the field of deep learning, better algorithms are constantly being proposed, and reference implementations of these algorithms are being released. Therefore, LIM Tracker has a plugin mechanism that allows users to call reference implementations written in the Python language as external programs and link them with this software to operate the DL training and recognition processes from this software. Users with programming knowledge can implement their own plug-ins to work with any DL algorithm. Currently, plugins are available to work with four different algorithm implementations (“Cellpose”^9^, “StarDist” , “Mask R-CNN”^24,25^, and “YOLACT +  + ”^26^ (Supplementary S2.4 to 2.7)). Each algorithm has different features, such as processing speed, recognition accuracy, and required GPU memory size. Users can apply them according to the specifications of their execution environment.

#### Hardware requirements

This software has been tested on Windows10 or Linux (Ubuntu18.04) using a PC with processor Intel Core i7-6700, 16 GB of RAM. Also, when using the DL recognition function, a GTX1080Ti 11 GB was used (we recommend using nvidia GPUs with 8 GB GPU memory or higher, CUDA 10.0 or higher).

## Results and discussion

### Case 1: NRK-52E/EKAREV-NLS

**Figure 4:**
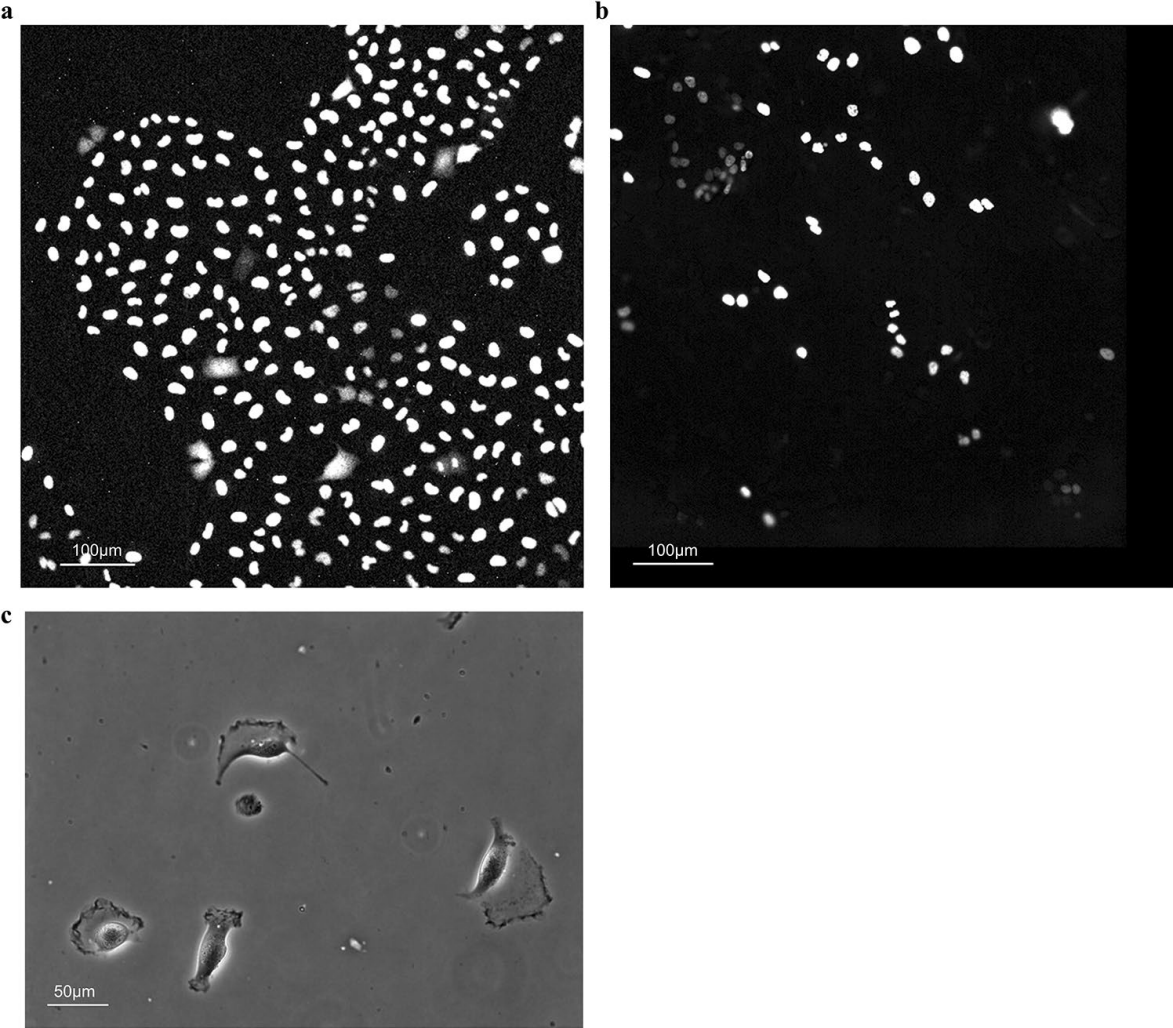
Test images. (**a**) NRK-52E/EKAREV-NLS. (**b**) MCF-10A/H2B-iRFP-P2A-mScarlet-I-hGem-P2A-PIP-NLS-mNeonGreen. (**c**) Glioblastoma-astrocytoma U373 cells on a polyacrylamide substrate (PhC-C2DH-U373).

**Figure 5:**
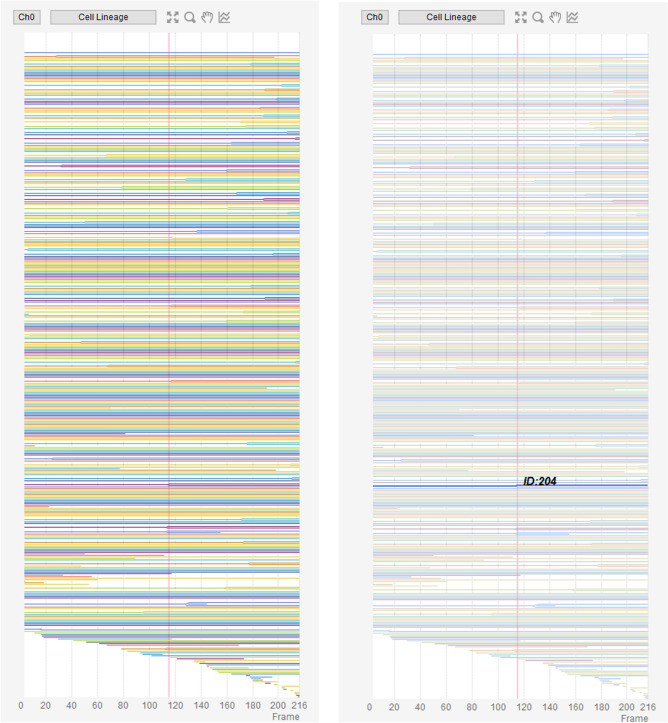
Cell lineage display. Cell lineage display of Case 1 [right: selected and highlighted lineage for a specific ROI (ID 204)].

**Figure 6:**
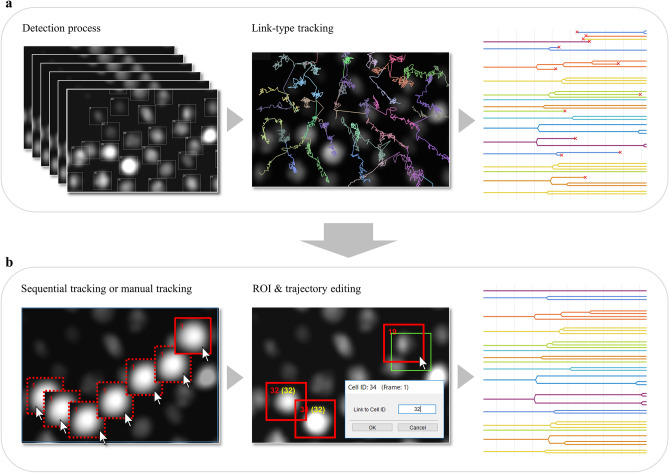
Conceptual diagram of the tracking process. (**a**) Conceptual diagram of the tracking process for an entire frame using link-type tracking. Depending on the detection accuracy and other factors, it may not always be possible to track accurately, but it is easy to detect erroneous tracking by displaying the cell lineage and checking for breaks and branches in the genealogy. (**b**) In addition to link-type tracking, it is possible to seamlessly combine sequential tracking, manual tracking, and ROI/trajectory editing functions. For example, sequential tracking can be used to add or modify trajectories only for mistracked areas, and accurate tracking results can be obtained efficiently through the combination.

**Figure 7:**
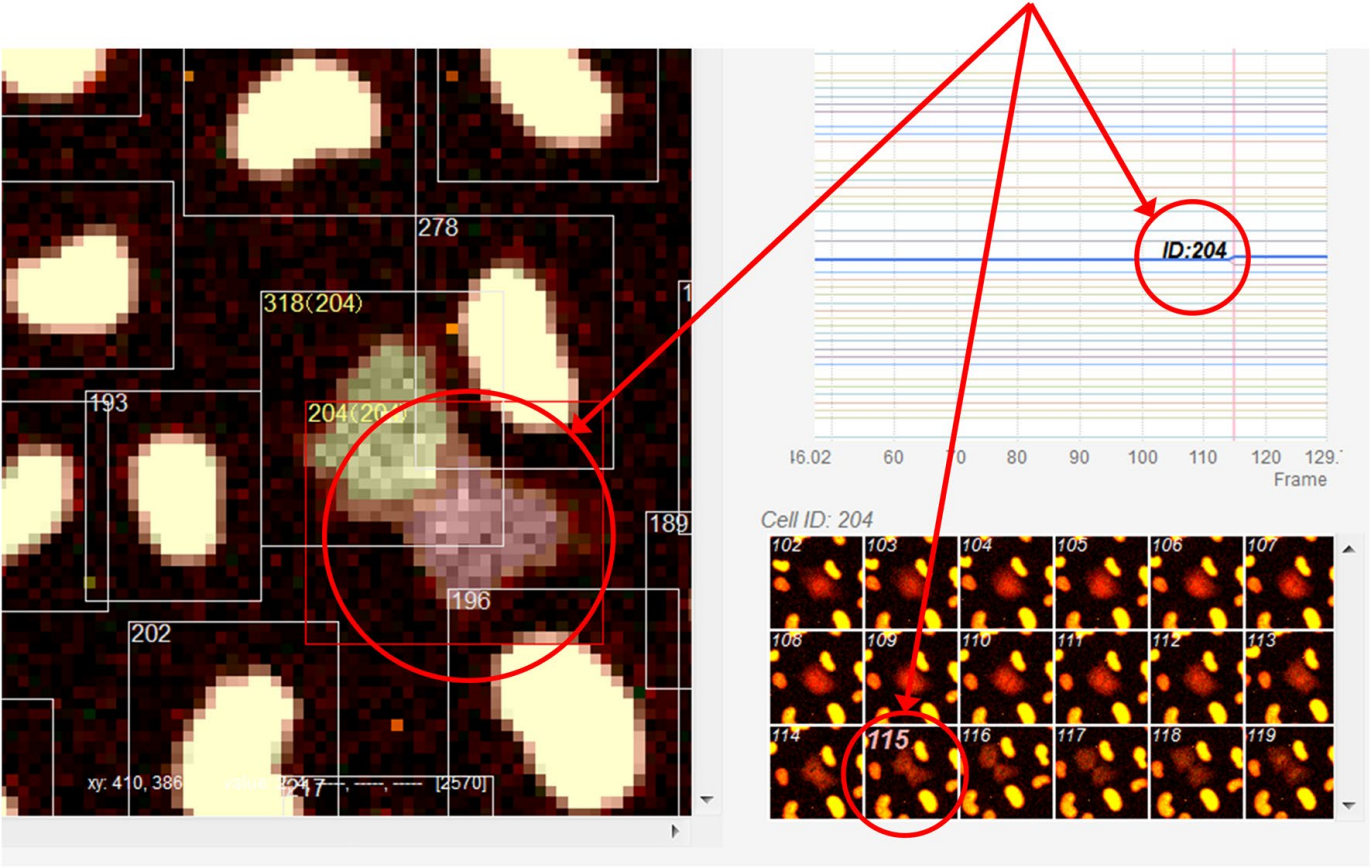
Example of this software’s screen display. A scene during cell division. Clicking on an ROI (ID 204) in the original image immediately displays the corresponding cell lineage and a montage of the image before and after the frame in question. Clicking on any position in the cell lineage will display the corresponding image frame, ROI, and montage image. Clicking on a montage image will generate the same response.

In this section, we introduce a tracking case study that combines the link-type tracking and sequential tracking functions, and the ROI and trajectory editing functions, given that the ease of such combinations is one of the characteristic features of LIM Tracker. The images used in this study were taken from NRK-52E/EKAREV-NLS cells derived from normal rat kidney epithelial cells and captured the stochastic changes in the activity of ERK molecules using a FRET biosensor. ERK is involved in cell proliferation, differentiation, and tumorigenesis^27^. It has been reported that cell proliferation is regulated by frequency modulation of the stochastic activation of ERK molecules^20,21^. It has also been reported that intercellular propagation of ERK activation determines the directionality of collective cell migration^28^. Thus, quantifying the dynamics of the activity of ERK molecules is an important technical challenge. Time-lapse images were acquired with an IX81 fluorescence microscope (Fig. 4a; Olympus; 757.76 μm/512 pixels, × 20 objective, Bin 4 × 4, 512 × 512 pixels, 217 frames, 80 s frame interval). In this dataset, there are about 300 cells in each frame, for a total of 64,181 cells in all 217 frames. Cell division occurs at 53 sites, and the number of cells gradually increases. Figure 5 shows an example of the cell lineage display. For these cells, the same cells are linked between images to create a trajectory. The cell population imaged by fluorescence microscopy can be recognized with relatively high accuracy by referring to the peak position of the brightness. Because of the large number of cells, it is efficient to apply the link-type tracking function to create most of the trajectories in this dataset first, and then apply the sequential tracking, manual tracking, and editing functions to correct the errors in some of the mis-tracked areas (Fig. 6). After applying the link-type tracking function, the areas of mis-detection or mis-tracking can be identified, based primarily on the cell lineage display. In the cell lineage, tracking errors often appear in the form of broken trajectories or unnatural branching (e.g., repeated division in a short period of time). By clicking on a lineage, the corresponding image frame and ROI are immediately recalled and displayed, making it easy to visually check for errors. In addition, if there is an ROI (trajectory) on the screen that the user wants to examine in detail, clicking on it will highlight all of the linked ROIs and related data and lineages, helping to visually understand the correctness of the link. Figure 7 shows an example of clicking on an ROI (ID number 204) on the original image and highlighting it. After applying the link-type tracking to this dataset, the tracking errors that were identified can be summarized into two main categories.

1. In the case of two cells moving in close contact, the individual cells cannot be divided accurately and are misidentified as a single cell, resulting in no proper linking.
2. Shape change (cell swelling) occurs during cell division, and over-segmentation (recognition of one cell as a plurality of cells) occurs, resulting in erroneous branching by judging that cell division has occurred.

For case (1), we reapply the sequential tracking function to each of the two cells to recreate the trajectory. When applying sequential tracking, the frame display is updated in real time as the tracking progresses, so the user can visually check the correctness of the destination and positional shift as the process proceeds. In situations where tracking is difficult and misalignment occurs, the process is canceled once, the misalignment is corrected using the mouse, and then tracking is restarted from the corrected position. By repeating this process, tracking can be performed efficiently. Depending on the closeness of the two cells, the sequential search may be difficult and misalignment may occur. In such cases, the manual tracking function can be useful. The tracking can be performed using both automatic and manual tracking as appropriate. For case (2), the over-segmented region (ROI) is deleted using the mouse and a new ROI is created. To create the shape of the region, an auxiliary function for automatically setting the shape at the same time as creating the ROI can be used. However, in some cases, the shape differs between before and after the division because of its irregularity, and an appropriate shape is not set; in this case, an arbitrary shape is set by tracing the boundary line of the region using the pen tool function. The link between the mother cell and daughter cell can be reconfigured individually from the pull-down menu of the newly created ROI. If there are many areas that need to be corrected, it is also possible to recreate all of the ROIs erroneously recognized in the whole frame in advance, and then reapply the link-type tracking function to relink the entire frame. Link processing in this function targets all ROIs located on an image, and can be executed at any arbitrary timepoint not only for ROIs generated by recognition processing but also for ROIs directly generated by a user’s mouse operation.

The above process enables accurate tracking results to be obtained efficiently. We measured the recognition and tracking performance immediately after applying the link-type tracking function (before correcting the tracking errors). The evaluation index is the one used in the ISBI Cell Tracking Challenge^6^, and is calculated using a publicly available evaluation program. The recognition accuracy (SEG), which is affected by the accuracy of the region shape, is based on the Jaccard similarity of the regions of agreement between the correct answer (Ground Truth) and the recognition result. The detection accuracy (DET) and the tracking accuracy (TRA) is a graph-based method that represents the cell lineage as a directed acyclic graph, and the agreement score is calculated by comparing the graph created as the correct answer with the graph of the tracking result. The recognition accuracy (SEG) was 0.96, the detection accuracy (DET) was 0.98 and the tracking accuracy (TRA) was 0.98, indicating that the tracking was relatively accurate. The recognition process (including the calculation of feature values) took 23 s (9.4 fps) for the entire 217 frames, and the link-type tracking took 5 s (43.4 fps).

In addition, sequential tracking, which was used to correct the errors in the link-type tracking in this case study, took an average of 17 s (12.8 fps) when one cell was selected individually and tracked through 217 frames. If the user do not need the morphological information of the region and only need to detect and track the spot position, the process is simpler because the user do not need to create the region shape. However, the basic procedure is the same as above.

### Case 2: MCF-10A/H2B-iRFP-P2A-mScarlet-I-hGem-P2A-PIP-NLS-mNeonGreen

Here, we present a case study using a dataset from the imaging of MCF-10A/H2B-iRFP-P2A-mScarlet-I-hGem-P2A-PIP-NLS-mNeonGreen cells derived from human mammary epithelial cells. The cells express the S/G_2_/M phase marker mScarlet-I-Gem^16^ and the G_1_/G_2_/M phase marker PIP-tag-NLS-mNeonGreen^17^, as well as H2B-iRFP, a nuclear marker required for tracking. The state of the cell cycle differs from one cell to another; therefore, quantifying the cell cycle at the single-cell level is important for understanding the molecular mechanisms underlying cell cycle. The image set studied in this case consists of time-lapse images taken by IXM-XLS (Fig. 4b; 697.68 μm/1080 pixels = 0.646 μm/pixel, × 20 objective, 1080 × 1080 pixels, 207 frames, 20 min frame interval). Each image is produced by the stitching together of multiple original images, and the cells differ markedly in size and luminance; some cells have extremely low luminance values that are the same level as the background. In this case, we followed the same procedure as in Case 1 for recognition and tracking. After applying the link-type tracking function, most of the tracking errors that could be confirmed at the time before the correction were due to the fact that the boundary between two cells was not detected when they were adjacent to each other, and they were misrecognized as a single cell, resulting in a break in the trajectory and incorrect branching. As in Case 1, we used the cell lineage display to find the mis-tracked area and correct the error. When misrecognition occurs over a long frame interval, it is effective to apply the sequential tracking function to individual cells that fail to be recognized. However, if the proximity is so close that tracking is difficult and misalignment occurs, it can be corrected by switching to manual tracking as appropriate. We measured the recognition and tracking performance immediately after applying the link-type tracking function (before correcting the erroneous tracking) using the same index as in Case 1. The recognition accuracy (SEG) was 0.90, the detection accuracy (DET) was 0.95 and the tracking accuracy (TRA) was 0.94. The recognition accuracy is lower than that of Case 1 because the boundary between adjacent cells is more ambiguous and there is no difference in luminance between them, making it difficult to segment them. The recognition process (including the calculation of feature values) took 46 s (4.5 fps) for the entire 207 frames, and the link-type tracking took 1 s (207 fps).

### Case 3: Phase contrast microscope image tracking

Finally, using the dataset “Glioblastoma-astrocytoma U373 cells on a polyacrylamide substrate (PhC-C2DH-U373)” published in the ISBI Cell Tracking Challenge^6^ (Fig. 4c), we introduce an example of tracking processing using the DL recognition function and link-type tracking processing. A cell imaged by a phase contrast microscope is one of the targets difficult to detect by conventional recognition processing because the cytoplasm is generally irregularly shaped and there is no difference in contrast with the background. In this case study, we applied the DL training and recognition functions. This software is equipped with a pen tool function as an annotation tool, which allows user to freely create mask regions on the image loaded for training image creation. In this study, we used the SilverTruth annotation image provided in the dataset above and converted it to the correct mask image format for the DL training process. Specific operation procedures for the DL training and recognition function are described below.

- Step 1: Load the original image as a training image into the software, and create the correct mask area by tracing the boundary line of the tracking target using the pen tool function. (In this case, the SilverTruth image is converted to the correct mask format and used instead.)
- Step 2: On the GUI, specify the destination of the weight file that will be the training result. In addition, if necessary, set hyper-parameters related to DL training (e.g., number of epochs).
- Step 3: Start training by pressing the button. When the training is completed, a weight file representing the training result is generated at the specified storage destination.
- Step 4: When performing DL recognition, the trained weight file is selected from the file dialog, and the DL recognition service program written in the Python language is started.
- Step 5: Read the image to be recognized on the software, and execute DL recognition processing in cooperation with a DL recognition service program.

After the DL recognition processing, the tracking processing by the link-type tracking function was executed. When the tracking error was confirmed using the cell lineage display, among others, only a few small fragment areas with long migration and unclear boundaries were not detected. The undetected region was added as a new ROI region using the pen tool function, and when the link-type tracking function was applied again, the correct tracking result was obtained. As a result of measuring the recognition performance and tracking performance immediately after the application of the link-type tracking function (before correcting the erroneous tracking) using the same indices as in Case 1, the recognition accuracy (SEG) was 0.93, the detection accuracy (DET) was 0.98 and the tracking accuracy (TRA) was 0.98, confirming that highly accurate tracking was possible. The DL recognition process (including the calculation of feature values) took 116 s (1.0 fps) for the entire 115 frames, and the link-type tracking took 0.4 s (230 fps).

Through Case 1 and Case 2, it was shown that accurate tracking results could be efficiently obtained by combining multiple tracking functions and cell lineage display, among others, incorporated into this software. Many existing software programs do not have sufficient means of checking the validity of the tracking results, and it is always unclear whether the correct data have been obtained after the automatic tracking process. Especially when there is a large number of tracked objects or these objects are dense, it is often difficult for software to check the correctness of individual trajectories. To overcome these issues, LIM Tracker has a UI that enables interactive operation and incorporates an excellent visualization means that displays data in real time. Even in the cases described above, the entire linked ROI group (trajectory) can be highlighted with one click of the mouse on the ROI located on the original image, and the correctness can be easily confirmed visually. Furthermore, it is possible to highlight a feature list, related data on a scatter plot, or a graph showing time-series feature changes based on trajectory information in conjunction with a click of the mouse on the ROI, and to call up and display a montage (group of thumbnails) image of the corresponding trajectory. Conversely, when an arbitrary coordinate on the time-series graph is clicked with the mouse, a corresponding image frame is called to highlight the ROI and update the montage image display. Even when the position or size of the ROI is corrected with the mouse, the feature quantity is immediately recalculated, and the related tables, scatter plot, graph, montage, and so on are updated in real time. These features are also incorporated into the cell lineage display, and the corresponding frame and ROI, among others, can be easily retrieved by simply clicking on the lineage as described in the above example.

In addition, among existing software, there are almost no options providing usable and practical correction methods for tracking results. For example, if the goal is to obtain 100% accurate and error-free tracking data, the only option is to abandon automatic tracking, which is not correctable, and use manual tracking. As introduced in the above example, LIM Tracker can perform sequential tracking, manual tracking, or both by limiting to erroneous tracking points found after automatic tracking by the link-type tracking function. By partially remaking the track, the erroneous points can be effectively corrected without reprocessing the whole, which greatly reduces the labor-intensiveness of the procedure. As another application of the combined approach, for example, when the object to be processed changes along the time axis and there is a frame section that is difficult to recognize on the way, a combination of changing the method for each section and tracking is possible. In addition, when the number of targets is small, a combination of utilizing manual tracking only in the section where automatic tracking is difficult is also possible, while utilizing sequential tracking in which highly precise processing is mainly possible.

Other features include simple and flexible editing of ROIs and trajectories based on direct manipulation with the mouse. For example, if misrecognition is found during the recognition process, it is easy to correct the over- or under-detected part using the mouse and then perform the linking process. In addition, if a link error is found after the linking process, it is easy to correct it by relinking directly with the mouse. Moreover, it was shown through case 3 that the latest DL recognition algorithm can be easily trained and recognized by the users themselves. In general, the recognition process using machine learning such as deep learning does not always work properly for user-specific datasets by simply using the default process; in such cases, the recognition process itself needs to be trained by the user. However, software incorporating existing DL recognition processing has poor functions related to DL training, and it is often difficult to construct processing optimized for a user’s own purpose. The standard DL training procedure consists of a series of steps, such as first creating a training image by annotation work for the image, then having the DL training algorithm learn the image, and then generating a training result, that is, a weight (model) file representing the learned network state. Our software enables users to perform the above steps simply and consistently without requiring expert knowledge of deep learning, machine learning, or other fields, and to obtain highly accurate tracking results by easily constructing the recognition process optimized for the desired data by users themselves.

The main challenges regarding the current state of LIM Tracker can be summarized in three points. First, there is a limitation in functional extensibility. Currently, there is a plugin mechanism for the default (non-DL) recognition and DL recognition processes, and users can incorporate any algorithm from outside the software; however, for the tracking process, users can only use the preloaded algorithms. In addition, the analysis result to be measured in this software can only use pre-defined features, and there is no extension mechanism to allow users to arbitrarily define the features that they want to output. Second, interoperability with other analytical software is limited. The output of analysis result in this software is limited to CSV text and image files, and there is no general-purpose interface to enable smooth data linkage from this software to existing more specialized analytical software (e.g., R, Matlab, etc.) or vice versa. Third, this software prioritizes support for Windows OS, and some of its functions are limited in Linux. We would like to improve the above points in the future.

## Conclusion

LIM Tracker is equipped with conventional tracking functions consisting of recognition and frame-to-frame link processing, pattern-matching-based sequential search tracking, and manual tracking functions, which can be seamlessly combined as appropriate to improve the efficiency of tracking operations. In addition, the DL recognition function can also be used to build highly accurate recognition processes for a wide range of objects. Training functions including annotation tools are built-in, and a series of DL operations from training to recognition can be performed by a simple procedure. Moreover, the software enables interactive operation of data display items (ROI, tables, graphs, thumbnails, etc.) on the screen to visually and intuitively evaluate the validity of tracking results, and has a function to select and highlight related items in real time using the mouse. It also has a simple and flexible mouse-based correction method in the case of mis-tracking. The application of this software is not limited to any particular subject but can support the quantification of a variety of dynamic phenomena in biological processes. By providing this software to the life science community, we hope to contribute to improving the efficiency of data analysis in time-lapse live imaging studies.

## Supplementary Information

Supplementary Information.

## Data Availability

The datasets for cases 1 and 2 are available at https://github.com/LIMT34/LIM-Tracker. The dataset for case 3 can be downloaded from the ISBI Cell Tracking Challenge website at http://celltrackingchallenge.net/2d-datasets/.

## Code availability

LIM Tracker is implemented as a plugin for ImageJ/Fiji. The software can be downloaded from https://github.com/LIMT34/LIM-Tracker.
