# Supplementary material for: LIM Tracker: a software package for cell tracking and analysis with advanced interactivity
Source: Sci Rep. 2022 Feb 17;12:2702. doi: 10.1038/s41598-022-06269-6 (PMC8854686; doi:10.1038/s41598-022-06269-6)
Supplement: Supplementary file 1 — Supplementary Information. [file 41598_2022_6269_MOESM1_ESM.docx]

**LIM Tracker: a software package for cell tracking and analysis with advanced interactivity**

Hideya Aragaki ^1,^*, Katsunori Ogoh ^1^, Yohei Kondo ^2,3,4^, Kazuhiro Aoki ^2,3,4^

^1^ Innovation and Core Technology Management, Olympus Corporation, Kuboyama 2-3, Hachioji, Tokyo 192-8512, Japan

^2^ Quantitative Biology Research Group, Exploratory Research Center on Life and Living Systems (ExCELLS), National Institutes of Natural Sciences, 5-1 Higashiyama, Myodaiji-cho, Okazaki, Aichi 444-8787, Japan

^3^ Division of Quantitative Biology, National Institute for Basic Biology, National Institutes of Natural Sciences, 5-1 Higashiyama, Myodaiji-cho, Okazaki, Aichi 444-8787, Japan

^4^ Department of Basic Biology, School of Life Science, SOKENDAI (The Graduate University for Advanced Studies), 5-1 Higashiyama, Myodaiji-cho, Okazaki, Aichi 444-8787, Japan

* Corresponding Author: [hideya.aragaki@olympus.com](mailto:hideya.aragaki@olympus.com)

**Supplementary information**

**S1 Introduction**

**S1.1. Comparison of the performance**

We use the dataset “Glioblastoma-astrocytoma U373 cells on a polyacrylamide substrate (PhC-C2DH-U373)” published in the ISBI Cell Tracking Challenge. In this study, we used the SilverTruth annotation image provided in the dataset above and converted it to the correct mask image format for the DL training process. The evaluation index is the one used in the ISBI Cell Tracking Challenge, and is calculated using a publicly available evaluation program. The recognition accuracy (SEG) , which is affected by the accuracy of the region shape, is based on the Jaccard similarity of the regions of agreement between the correct answer (Ground Truth) and the recognition result. The detection accuracy (DET) and the tracking accuracy (TRA) is a graph-based method that represents the cell lineage as a directed acyclic graph, and the agreement score is calculated by comparing the graph created as the correct answer with the graph of the tracking result. For the recognition process in TrackMate, we used “Stardist (Stardist detector custom model)”. However, since it does not have a training function, we created a trained weight file by command line operations. The parameters set for training are stepperepoch = 4 and epochs = 10000. For training “Mask R-CNN” used in Usiigaci's recognition process, we used the Python script file (Usiigaci/Mask R-CNN/train.py) available in official repository, and created a trained weight file based on command line operations. We used “Mask R-CNN” for the LIM Tracker recognition process, and the training function (integrated UI including annotations) allows for efficient training. The parameters set in the UI for the training function are Step per epoch = 100, Number of epochs = 400, Backborn network = resnet101.

**S2 Methods**

**S2.1. Linear Assignment Problem (LAP) algorithm**

LAP algorithm first calculates a cost, such as distance, for every possible cell-to-cell combination (link) between adjacent frames, and creates a cost matrix defined using all of the costs. Then, by applying an efficient optimization method such as the Hungarian method to the cost matrix and estimating the combination in which the total sum of costs becomes the smallest, the optimum combination of links can quickly be specified. This is performed between all adjacent frames to generate the trajectories.

**S2.2. Particle Filter**

Particle filter is a method to obtain the probability distribution of the existence of a tracking target by discrete approximation of the target’s position based on a large number of particles representing the candidate positions to be moved. It consists of iterations of prediction and update steps, estimating the position of each particle in the next frame, obtaining the likelihood that represents the probability of the existence of the tracking target for each of those particles, and estimating the state of the target (the destination position) from the likelihood distribution.

**S2.3. Interactive real-time data linkage display**

In LIM Tracker, a unique ID number is always clearly indicated on the upper left of the ROI, and the same ID number is given to the ROI indicating the same target across frames so that the connection of the trajectory can be uniquely confirmed on the screen. Various feature quantities and trajectory information measured for each ROI are displayed on the screen as multiple data display items, such as tables, scatter plots, graphs, and montage images, which are linked by ID numbers. However, in the presence of a large amount of data, it is not easy to grasp the relationship among the data by simply arranging them in a fixed manner on the screen. This software incorporates the following functions to support the user’s intuitive understanding of various types of data. First, to enable the user to always check the latest status of tracking on the screen, there is a function for performing feature measurement and acquisition of trajectory information regarding the ROI immediately at the time when a change occurs in the state of the ROI. In other words, this occurs after recognition/tracking processing or after the ROI is operated with a mouse, and the results are represented in real time in various displays on the screen. Second, to allow the user to intuitively select the ROI and data of interest, there are functions enabling the user to directly choose the ROI and various data items displayed on the screen by clicking the mouse. Third, to minimize the burden of searching for data, there is a function to automatically select and highlight all related data linked by an ID when specific data are selected on the screen. The combination of the above enables interactive operability using the mouse for the ROI and various data display items, and when the user has an object of interest on the screen, all data related to it can be quickly called up by direct manipulation.

**S2.4. Cellpose**

Cellpose is a cell-image-specific instance segmentation algorithm proposed by Stringer et al. The trained models based on large datasets are available online in advance, and can be downloaded and used to perform highly accurate recognition for a wide range of target types. This software works with the PyTorch implementation available in the official repositories, and can conduct training and recognition processes on its own dataset. Cellpose generates two types of gradient map, one for each of the XY directions extending from the cell center to the boundary, obtained by thermal diffusion simulation, and a foreground map to separate the background and foreground, and performs training based on the U-Net architecture. In recognition, each gradient map and foreground map is estimated from the target image, and each instance region is recovered based on the flow field predicted by these maps.

**S2.5. StarDist**

StarDist is a cell-image-specific instance segmentation algorithm that predicts object probabilities and star-convex polygons parameterized by the radial distances. It is based on a simple U-Net architecture, it can detect circular objects with high accuracy.

**S2.6. Mask R-CNN**

Mask R-CNN is one of the state of the art (SOTA) in the field of instance segmentation, and achieves highly accurate recognition. This software can use the training and recognition process linked with the TensorFlow & Keras implementation provided by Matterport Inc. 16. Mask R-CNN involves two steps. In the first step, the feature map generated through convolutional operations by a backbone network [such as ResNet with Feature Pyramid Network (FPN) structure] is scanned by Region Proposal Network (RPN) to estimate the candidate regions that are likely to contain the target. In the second step, class identification, bounding box, and region mask are generated for each estimated candidate region, and high-precision mask regions are generated by correcting the misalignment of the bounding box using the RoiAlign process.

**S2.7. YOLACT++**

YOLACT++ is a SOTA in the field of “real-time” instance segmentation, which is slightly less accurate than Mask R-CNN, but can be run on relatively low specification GPUs. YOLACT++ is a single-stage method that simultaneously performs region extraction and category identification in a single network, and incorporates the concept of a “prototype mask” (equivalent to a dictionary mask = vocabulary or code-book) to estimate the target. Based on the feature map generated by the backbone network, a set of prototype masks and mask coefficients (the contribution of each prototype to the target region) are learned so that an arbitrary target region can be represented as a linear sum of k prototype masks.
